# Enhanced recovery after surgery society’s recommendations for liver surgery reduces non surgical complications

**DOI:** 10.1038/s41598-025-86808-z

**Published:** 2025-01-29

**Authors:** Robert Oehring, Eriselda Keshi, Karl-Herbert Hillebrandt, Pia F. Koch, Matthäus Felsenstein, Simon Moosburner, Wenzel Schöning, Nathanael Raschzok, Johann Pratschke, Jens Neudecker, Felix Krenzien

**Affiliations:** 1 Department of Surgery, Campus Charité Mitte and Campus Virchow-Klinikum, Charité-Universitätsmedizin, corporate member of Freie Universität Berlin, Humboldt-Universität zu Berlin, 13353 Berlin, Germany; 2 Berlin Institute of Health (BIH), Berlin, Germany; 3 Department of Surgery, Harzklinikum D.C. Erxleben, Quedlinburg, Germany

**Keywords:** Enhanced recovery after surgery (ERAS), Liver surgery, Complications, Biliary tract cancer, Liver cancer, Medical research, Hepatology, Surgical oncology

## Abstract

Enhanced Recovery after Surgery (ERAS) is a multimodal approach to improve surgical outcome and has been implemented in many fields of surgery in an international scale. The aim of this study was to evaluate the effect of the Enhanced Recovery after Surgery (ERAS) society recommendations in liver surgery and the impact on general and surgery-related complications. 1049 patients who underwent liver surgery from July 2018 to October 2023 were included. The ERAS program strictly followed the official ERAS society recommendations. As a control group (Non-ERAS) 90 patients were treated according to the clinic standard, while 959 patients were treated according within the ERAS measures. After propensity score (PSM) matching 87 Non-ERAS and 258 ERAS patients were analyzed by complications and cumulative sum analysis (CUSUM). ERAS implementation resulted in a significant decrease in general complications (control 27.6% vs. ERAS 16.3%, *p* = 0.033), largely attributed to a reduction in infection-related complications (control 20.7% vs. ERAS 9.7%, *p* = 0.007). When examining surgery-related complications no significant disparities were observed (control 17.2% vs. ERAS 17.1%, *p* = 0.968). The CUSUM analysis of general and non-surgical complications showed that the full effect of the ERAS program only became apparent after several years. Moreover, adherence increased over time consecutively from 62.5 to 72.5% in 4 years. The ERAS society recommendations for liver surgery reduced general complications but did not have any effect on surgery related complications. The effect of the ERAS program progressively improved over the years, highlighting the need for continuous effort to maintain and further enhance outcomes.

**Supplementary Information:**

The online version contains supplementary material available at 10.1038/s41598-025-86808-z.

## Introduction

Enhanced Recovery after Surgery (ERAS) is a multimodal approach to improve surgical outcome and has been implemented in clinical daily routine in many hospitals^1^. The ERAS protocol for different surgery consists of different items with different levels of evidence^2,3^. For liver surgery the ERAS society published the first guideline in 2016^4^ and updated them recently^2^. The guidelines have been prospectively validated for the first time by our group, demonstrating a reduction of complications graded by Clavien-Dindo^5^.

Indeed, in ERAS studies most complications are reported by Clavien-Dindo scale or Comprehensive Complication Index within ERAS studies. Using these standardized scales renders data more comparable within patient groups and between patient cohorts. However they fail to depict the effect of ERAS on specific complications such as bile leakage or anastomosis insufficiency, or non-surgical factors such as kidney failure, pneumonia, urinary tract infection, or wound infection^6^. Data about the specific effects would enable to improve the current recommendations according to the guidelines from the ERAS society, which are measures before, during and after liver resection^2^. Therefore the most common liver surgery specific complications were chosen to be analyzed. They were predetermined by the ERAS Interactive Audit System (EIAS) (Encare, Stockholm, Sweden). For example bile leakage is one of the most common and severe complications after liver surgery ranging between 4–17%^7,8^. Acute kidney failure can be associated with post-hepatectomy liver failure and is therefore also a good marker for postoperative complications^9^. The development of ascites has been observed to be associated with hepatic surgery and is commonly encountered in patients exhibiting significant hepatic dysfunction or cirrhosis^9^. Pleural effusion can also be related to the direct manipulation of the diaphragma in liver surgery^10^. Pneumonia, urinary tract infections, and wound infections are among the most common postoperative complications. However, these infections are not directly attributable to liver surgery. Consequently, these infections require meticulous monitoring and treatment.

The impact of the ERAS system on complications such as urinary tract infections, pneumonia, and thrombosis is a plausible consideration. Early mobilization, on the one hand, has been shown to enhance lung ventilation and improve blood flow, which may potentially prevent pneumonia and thrombosis. Furthermore, the early removal of the urinary bladder catheter can be associated with a reduced incidence of urinary tract infections.

Therefore, the aim of the present observational study was to validate the ERAS society recommendations for liver surgery according to a stratified classification system which divides complications into surgical and non-surgical and analyzes specific complications. In addition, the effect of the ERAS program will be shown over time.

## Methods

This prospective study received ethical approval from the Charité-Universitätsmedizin Berlin’s ethics committee under the application numbers EA2/108/18 and EA4/153/18 and was registered with the German Clinical Trials Register (DRKS00030908). Prior to their inclusion in the study, all participants provided written informed consent. The study was conducted in accordance with the Declaration of Helsinki (2013). The study enrolled patients undergoing elective liver surgery within an ERAS program at the Department of Surgery, Campus Virchow-Klinikum, Charité – Universitätsmedizin Berlin, spanning from July 2018 to October 2023. As a control group, 90 patients received treatment according to the clinic’s standard procedures between July 2018 and February 2019. Subsequently, from March 2019 to October 2023, a total of 959 patients underwent treatment according to the liver guidelines of the ERAS society^2^.

The ERAS program strictly followed the official ERAS society recommendations. The ERAS program is run by a core team of surgeons, anesthesiologists and specialized ERAS nurses. An ERAS team meeting is held once a week. Patients are also informed by the ERAS nurses before the operation. During the patient’s hospital stay, ERAS nurses conduct specialized ERAS visits to the patient’s ward. Patient data for our study was entered into the ERAS Interactive Audit System (EIAS), which is scientifically supervised by the ERAS Society. Since the inception of the ERAS program, all patient data has been consistently uploaded to this system. EIAS offers predefined categories alongside their respective subcategories, allowing for classifications such as: Compliant: Adhering to ERAS guidelines, Non-compliant: Not following the ERAS protocol or Missing: Data not available or not entered. Complications were put into a given table. Analysis was done using the given classifications which were provided by EIAS. A compliance score was calculated for each ERAS item based on the compliance of each individual patient. The overall compliance score was then calculated from the compliance data of the ERAS items. This was based on the manually entered data in the ERAS system. Table 1, listing the ERAS items implemented at our institution, has been included in the document. Each item adheres strictly to the ERAS guidelines of the ERAS society^2^.

**Table 1:** Compliance items by care element.

Pre admission	Pre operative	Intra operative	Post operative
Preoperative nutritional status assement	Preoperative oral carbohydrate treatment	Skin preparation used	Postoperative glycaemic control
Preoperative nutritional treatment	Thrombosis prophylaxis	Avoiding hypothermia	Duration of IV fluid infusion (nights)
Smoking cessation	Preoperative sedative medication	Regional analgesia for open surgery	Weight change on POD1
Alcohol cessation	PONV prophylaxis administered	Use of omentum flap for left-sided liver	Postoperative artificial nutrition and early oral intake
Preadmission patient education	Pre-operative steroid administration	Use of 0.9% NaCl	Mobilisation at all on day of surgery
Preoperative biliary drainage	Antibiotic prophylaxis before incision	Prophylactic abdominal drainage	Mobilisation on postoperative day 1
		Avoidance of nasogastric tube	Mobilisation on postoperative day 2
			Mobilisation on postoperative day 3
			30 day follow up performed

### Inclusion criteria

Patients who were at least 18 years old, underwent elective liver surgery and provided informed consent were included.

### Exclusion criteria

All patients who did not meet the inclusion criteria and did not give informed consent.

### Complication grading

#### Surgical and non-surgical complications

Complications will be categorized into surgical (Table 2), directly related to the procedure performed and non-surgical complications (Table 3). This categorization was adopted from the ERAS^®^ Interactive Audit System (EIAS). The main aim of introducing such a classification is to provide more detail on the impact of an ERAS program on the different complications.

**Table 2:** Complications directly related to the surgery.

General surgical complication	Anastomotic leakage
	Urinary tract injury
	Mechanical bowel obstruction
	Postoperative paralytic ileus
	Deep wound dehiscence (SSI 2)
	Intraoperative hemorrhage
	Postoperative hemorrhage
Liver surgery complication	Bile leakage, biloma (SSI 3)
	Post-hepatectomy liver failure
	Ascites
	Delayed gastric emptying

**Table 3:** General complications.

Main	
Cardiovascular	Acute myocardical infarction, heart failure, cerebrovascular lesion, deep venous thrombosis, cardiac arrhythmia, cardiac Arrest, pulmonary Embolism
Respiratory system	Pneumonia, Pleural fluid, Pneumthorax, Lobar atelectasis, Respiratory failure
Urinary tract/renal	Urinary tract infection, Renal dysfunction, Urinary retention
Gastrointestinal	Pancreatitis, Vomiting, Diarrhea, Obstipation, Gastrointestinal haemorrhage
Infectious	Wound infection (SSI 1), Intra- or retroperitoneal abscess, Sepsis, Septic shock, Infected graft or prosthesis, Cholangitis

### Statistics

Descriptive statistics and data analysis were carried out using IBM SPSS Statistics (Version 29.0.1.1). Descriptive statistics are presented as mean ± standard deviation (SD) or number (n) and percentage (%). Patients were assigned to the Non-ERAS and ERAS group. For metric variables Welch t-test and for nominal variables Chi2-test or exact Fisher-test were performed. The significance level (α-level) chosen was 0.05.

Propensity score matching (PSM) was carried out using R studio (Version 2023.06.1 + 524) The score was calculated using a logit model (package “MatchIt”) based on the following parameter: age, gender, diabetes mellitus, serum bilirubin, total and surgical approach. The “Nearest Neighbor” method was used and a ratio = 3 with a caliper of 0.2 was applied.

A Cumulative Sum (CUSUM) analysis was performed to examine the course of complications over the process of implementing the ERAS program. The patients were sorted chronologically by appearance of a complication and were plotted on a chart from left to right. The number of complications were counted. Zero meant no complication and served as an orientation measure. Complications added up to a negative score. The ordinate (Y value) represents the cumulative deviation of the complications score from the series’ mean, arranged in chronological order, while the abscissa (X value) signifies the progression of time. The turning point for complications was identified by locating the nadir in the smoothed data.

## Results

### Patients characteristics

Table 4 shows the baseline characteristic of the cohort before and after PSM. A total of 1049 patients were included, with 90 patients serving as the control group. These control patients (Non-ERAS) received the standard clinic protocol for liver surgery before the implementation of the ERAS program. A total of 959 patients were included in the ERAS group. No significant differences were observed in age, sex, preoperative weight, or height. Laboratory values for hemoglobin and leukocytes also showed no statistical differences, except for bilirubin, which was significantly different (*p* = 0.004). Also no statistical differences were observed for the presence of diabetes mellitus, neoadjuvant radiotherapy to the operation field, neoadjuvant chemotherapy and length of stay (*p* > 0.05). After 1:3 PSM, the Non-ERAS group consisted of 87 patients, whereas the ERAS cohort comprised 258 patients, forming the PSM cohort for subsequent analysis. In this cohort, no statistically significant differences were found in all observed baseline characteristics. Also bilirubin levels were equal after matching. Therefore groups are balanced after PSM showing no relevant difference in baseline characteristics.

**Table 4:** Baseline characteristics of the total cohort and PSM cohort.

	Total cohort			PSM 1:3		
	Non- ERAS	ERAS	*p*	Non- ERAS	ERAS	*p*
Parameter						
Total n	90	959		87	258	
Female	37 (41.1)	436 (45.5)	0.363	36 (41.4)	107 (41.5)	0.988
Age (years)	63.4 ± 13.8	61.5 ± 13.2	0.204	63.1 ± 13.9	62.7 ± 13.1	0.799
Preoperative weight (kg)	75.5 ± 15.6	77.3 ± 17.1	0.339	75.6 ± 15.7	77.6 ± 17.9	0.365
Height (cm)	171.2 ± 8.8	172.2 ± 9.3	0.322	171.4 ± 8.6	172.2 ± 9.2	0.479
Serum bilirubin total (mg/dl)	0.67 ± 1.1	0.47 ± 0.59	**0**.**004**	0.68 ± 1.1	0.56 ± 0.76	0.295
Hemoglobin (g/dl)	13.2 ± 1.7	13.2 ± 1.7	0.738	13.2 ± 1.7	13.1 ± 1.8	0.668
White blood cell count	7.4 ± 3.6	7.2 ± 2.6	0.574	7.4 ± 3.7	7.5 ± 2.5	0.852
Diabetes mellitus	20 (23)	157 (16.4)	0.184	19 (21.8)	61 (23.6)	0.771
Neoadjuvant chemotherapie	26 (29.9)	352 (36.7)	0.317	26 (29.9)	93 (36)	0.296
Neoadjuvant radiotherapy to operating field	2 (2.3)	23 (2.4)	0.907	2 (2.3)	11 (4.3)	0.529
LOS (night at hospital after primary operation)	10.3 ± 8.6	10.3 ± 14.9	0.993	10.2 ± 8.7	10.4 ± 12.3	0.900

The data are presented as mean ± SD or n (%).

Siginficance value is bold.

### Surgical characteristics after PSM

Table 5 provides a comprehensive overview of the procedures performed in both groups after PSM. Surgical approach and type of surgery showed no significant difference between the control and ERAS cohort (*p* > 0.05). There was no difference in the type of surgery either. Segmentectomy emerged as the most frequently performed liver surgery in both cohorts, constituting 47.1% in Non-ERAS and 39.1% in ERAS. Venous reconstruction (3.4% in Non-ERAS vs. 1.9% in ERAS, *p* = 0.681) were more frequently performed in the ERAS group whereas hepaticojejunostomy (6.9% in Non-ERAS vs. 11.6% in ERAS, *p* = 0.212) were more frequently performed in the ERAS group, but without significance for both.

**Table 5:** Surgical characteristics Non-ERAS and ERAS after PSM.

Parameter	Non-ERAS	ERAS	*p*
Surgical approach			0.396
Open	34 (39)	110 (42.6)	
Laparoscopic	39 (44.8)	107 (41.5)	
Robotic	11 (12.6)	33 (12.8)	
Converted from minimal invasive to open	3 (3.4)	8 (3.1)	
Type of surgery			0.29
Exploration only	5 (5.7)	14 (5.4)	
Left hemihepatectomy	13 (14.9)	26 (10.1)	
Extended left hemihepatectomy	7 (8)	16 (6.2)	
Right hemihepatectomy	8 (9.2)	26 (10.8)	
Extended right hemihepatectomy	8 (9.2)	42 (16.3)	
Segmentectomy	41 (47.1)	101 (39.1)	
Wedge resection or minor resection	5 (5.7)	26 (10.1)	
Other	0	7 (2.7)	
Venous reconstruction	3 (3.4)	5 (1.9)	0.681
Hepatikojejunostomie	6 (6.9)	30 (11.6)	0.212

The data are expressed in numbers and percentages (%).

### Surgery related complications after PSM

In total there were no statistically differences in surgery related complications after PSM when comparing the ERAS group (17.1%, *n* = 44) to the Non-ERAS group (17.2% (*n* = 15), *p* = 0.968 (Table 6). Liver specific complications were less frequent compared to the control group, while not reaching significance. No significant variances were observed for bile leakage and biloma between the two groups, whereas ascites occurred more frequently in the control group with statistical significance (*p* = 0.037). Additionally, a slightly higher occurrence of anastomotic leakage was noted in the control group, although statistical significance was not attained. Postoperative paralytic ileus, deep wound dehiscence, intra- and postoperative hemorrhage were not different between both groups. Urinary tract injury as well as mechanical bowel obstruction, Delayed gastric emptying or intraoperative excessive hemorrhage were not present in either cohort. Taken together, no significant difference was observed between the control and ERAS group concerning surgery-related complications.

**Table 6:** Surgery related complications after PSM.

	PSM		
	Non-ERAS	ERAS	*p*
Surgery related complications (patient with at least one complication)			
	15 (17.2)	44 (17.1)	0.968
Liver surgery complication			
Total	11 (12.6)	30 (11.6)	0.800
Bile leakage	*5 (5.7)*	*20 (7.8)*	*0.533*
Post-hepatectomy liver failure	*1 (1.1)*	*6 (2.3)*	*0.684*
Ascites	*4 (4.6)*	*2 (0.8)*	***0.037***
Biloma	*4 (4.6)*	*9 (3.5)*	*0.745*
Delayed gastric emptying	*0*	*0*	
General surgery complication			
Total	5 (5.7)	20 (7.8)	0.533
Anastomotic leakage	3 (3.4)	*3 (1.2)*	*0.171*
Postoperative paralytic ileus	*0*	*3 (1.2)*	*0.575*
Deep wound dehiscence	*0*	*7 (2.7)*	*0.191*
Postoperative excessive hemorrhage	*2 (2.3)*	*5 (1.9)*	*1.000*
Other	*1 (1.1)*	*2 (0.8)*	*1.000*
Urinary tract injury	*0*	*0*	
Mechanical bowel obstruction	*0*	*0*	
Intraoperative excessive hemorrhage	*0*	*0*	

The data are expressed in numbers and percentages (%). Some patients suffered multiple complications.

Siginficance vaules are bold, Italic.

### Non-surgical complications after PSM

A significant reduction in general (non-surgical) complications (Table 7) was observed in the ERAS group compared to the control group (16.3% vs. 27.6%, *p* = 0.033). This difference was primarily driven by the presence of infectious complications (*p* = 0.007). Specifically, more superficial wound infections (SSI 1; control 10.3% vs. ERAS 4.7%, *p* = 0.055) and urinary infections (control 5.7% vs. ERAS 0%, p = < 0.001) were observed in the control group. Cardiovascular complications occurred almost similarly in both groups (control 2.3% vs. ERAS 2.7%, *p* = 0.201) which was also true for renal, pancreatic, and gastrointestinal complications (control 6.9% vs. ERAS 5.8%, *p* = 0.715). Cardiovascular complications were primarily attributed to thrombosis (control 2.3% vs. ERAS 0.8%, *p* = 0.265), with no major cardiovascular complications such as acute myocardial infarction, heart failure, cardiac arrest or pulmonary embolism occurring in either group. The incidence of renal dysfunction was similar between the groups (control: 3.4% vs. ERAS: 3.5%); however, urinary retention was more prevalent in the control group (control: 2.3% vs. ERAS: 0%, *p* = 0.063). Conversely, respiratory complications were less prevalent in the control group (control 1.1% vs. ERAS 5.8%), although this difference did not reach statistical significance (*p* = 0.082). The increased incidence in the ERAS group was mainly due to the presence of pleural fluid (control 0% vs. ERAS 3.9%). Additionally, no significant difference was observed in the occurrence of pneumonia (control 1.1% vs. ERAS 1.6%). No pneumothorax or atelectasis occurred in either group.

**Table 7:** Postoperative complications not directly related to the procedure after PSM.

	PSM		
	Non- ERAS	ERAS	*p*
Non-surgical complications (patient with at least one complication)			
	24 (27.6)	42 (16.3)	**0.033**
Cardiovascular			
Total	2 (2.3)	7 (2.7)	0.201
Cerebrovascular lesion	*0*	*3 (1.2)*	*0.575*
Deep venous thrombosis	*2 (2.3)*	*2 (0.8)*	*0.265*
Pulmonary embolus	*0*	*1 (0.4)*	*0.712*
Other	*0*	*1 (0.4)*	*1.000*
Heart failure	*0*	*0*	
Acute myocardial infarction	*0*	*0*	
Cardiac arrhythmia	*0*	*0*	
Cardiac arrest	*0*	*0*	
Respiratory system			
Total	1 (1.1)	15 (5.8)	0.082
Pneumonia	*1 (1.1)*	*4 (1.6)*	*1.000*
Pleural Fluid	*0*	*10 (3.9)*	*0.071*
Respiratory failure	*0*	*4 (1.6)*	*0.576*
Pneumothorax	*0*	*0*	
Atelectasis	*0*	*0*	
Renal, Pancreatic and Gastrointestinal			
Total	6 (6.9)	15 (5.8)	0.715
Renal dysfunction	*3 (3.4)*	*9 (3.5)*	*1.000*
Urinary retention	*2 (2.3)*	*0*	*0.063*
Gastrointestinal hemorrhage	*2 (2.3)*	*4 (1.6)*	*0.644*
Hepatic dysfunction	*0*	*1 (0.4)*	*1.000*
Obstipation or diarrhea	*0*	*2 (0.8)*	*1.000*
Other	*0*	*2 (0.8)*	*1.000*
Pancreatitis	*0*	*0*	
Infectious complications			
Total	18 (20.7)	25 (9.7)	**0.007**
Wound infection	*9 (10.3)*	*12 (4.7)*	*0.055*
Urinary tract infection	*5 (5.7)*	*0*	***< 0.001***
Intra- or retroperitoneal abscess	*1 (1.1)*	*6 (2.3)*	*0.684*
Sepsis	*1 (1.1)*	*1 (0.4)*	*0.441*
Septic Shock	*0*	*3 (0.2)*	*0.575*
Cholangitis	*1 (1.1)*	*1 (0.4)*	*0.441*
Increase in infection parameters without clear focus	*3 (3.4)*	*3 (1.2)*	*0.171*
Infected graft or prosthesis	*0*	*0*	

The data are expressed in numbers and percentages (%). Some patients suffered multiple complications.

Siginficance vaules are bold, Italic.

### Effect of the ERAS program over time

**Fig. 1:**
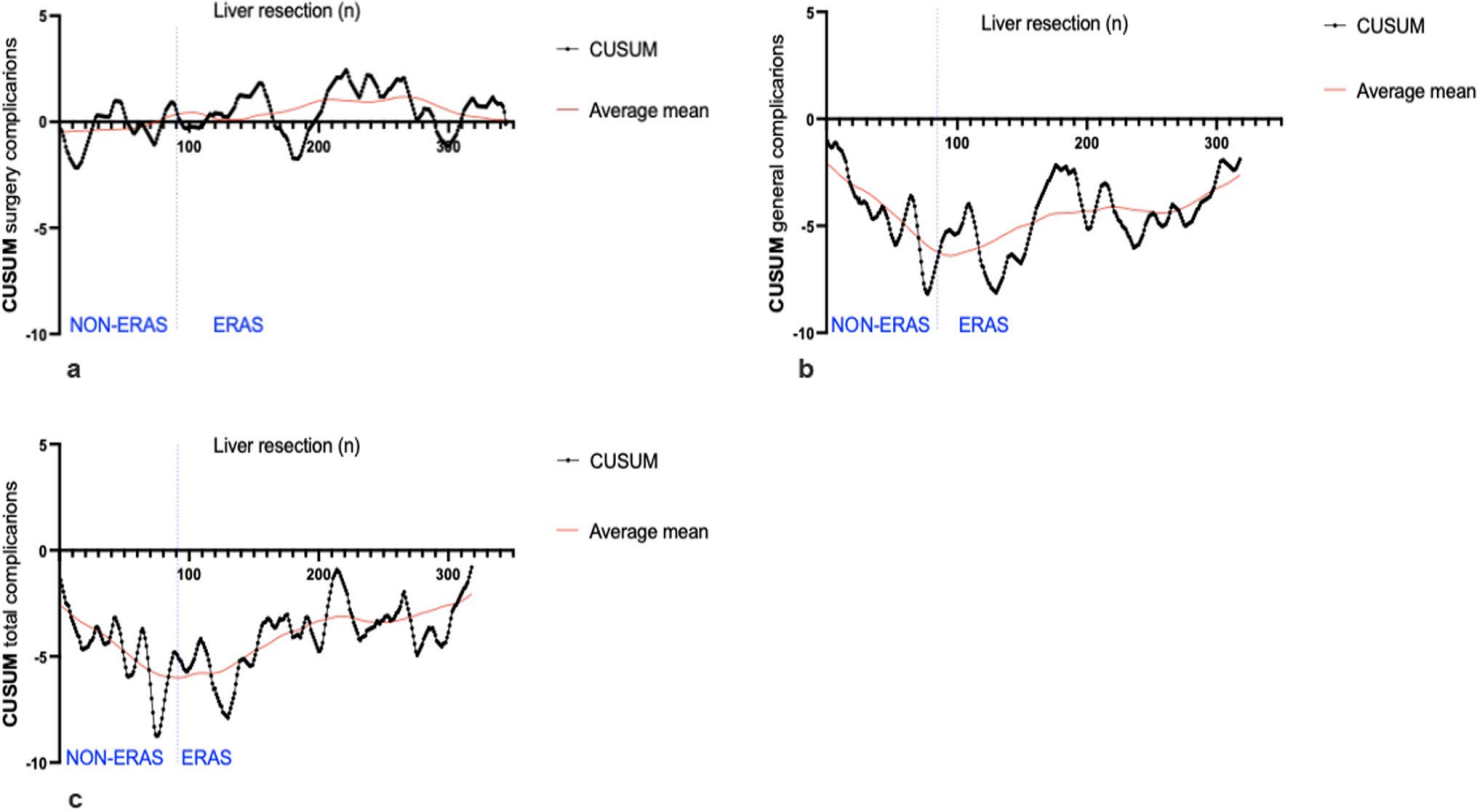
**a**-**c** CUSUM score of complications after PSM: (**a**) surgery complications, (**b**) general complications, (**c**) total complications; CUSUM analysis number of complications, average mean = solid red line.

Next, a CUSUM analysis was performed for general and surgery related complications (Fig. 1a-c) for PSM patients. For surgical complications (Fig. 1a), no clear trend was observed between non-ERAS and ERAS patients. As expected, an accumulation of complications and fluctuations in the CUSUM curve were evident reflecting clinical day life. For non-surgical complications (Fig. 1b) the curve first decreases for Non-ERAS patients implicating more complications than in average. while the curve increased after the implementation of the ERAS program, indicating a decline in complications A similar trend was also observed for the overall sum of all complications (Fig. 1c).

It can be observed, achieving a high level of compliance for each item is a significant challenge (Suppl. 1). Prior to admission, patients receive comprehensive education and assessment of their nutritional status. However, there is a notable lack of adherence to smoking and alcohol cessation. With regard to preoperative items, adherence exceeded 70% for all items except thrombosis prophylaxis. However, there are medical reasons why this should be omitted. Antibiotic prophylaxis before incision reached 99%. Intraoperative compliance varies, but postoperatively, adherence was high, especially for oral intake and mobilization, with the exception of mobilization on the day of the operation. Compliance increases over the study period. Starting at 62.5%, the adherence rate gradually rises to 72.5%. In contrast to increasing compliance, a decrease in the length of hospitalisation can also be observed (Suppl. 2).

The CUSUM analysis and adherence trend suggest that the establishment of an ERAS program requires years to improve compliance, LOS and postoperative complications. Over time, the impact of the ERAS program on reducing complications appears to increase, indicating that it takes a sustained effort to fully realize the benefits of this perioperative approach.

## Discussion

The aim of this study was to investigate which particular complications are changed by an ERAS program according to the ERAS guidelines. Numerous studies claim to have an ERAS program; however it is important to note that the ERAS items and adherence cut-offs vary significantly from study to study, making comparison nearly impossible. This inconsistency primarily arises because almost all studies do not follow the established guidelines set forth by the ERAS society^6^. Our research group has validated for the first time the ERAS guidelines recommendation previously, likewise a detailed analysis on single complications was still missing^5^. Although classification systems such as the Clavien-Dindo classification have been used to describe complications when evaluating the use of an ERAS system, we introduced a different system for evaluating complications in an ERAS programme for liver surgery. This could help to modify the current ERAS guidelines and improve patient outcomes. Note, the frequently used Clavien-Dindo classification or CCI categorizes surgical complications, while it does not give any information, whether for example, SSI or pneumonia are changed by an ERAS program. In the present study, the current ERAS guidelines of the ERAS society therefore have been validated according to their impact on surgery-related and non-surgery-related complications in detail.

Our results are in line to several meta-analyses on ERAS in liver surgery, which have shown that ERAS in liver surgery reduces the overall complication rates^6,11,12^, but with no data on which complications are modified. Our study aims to fill this gap in the literature by providing a detailed analysis of how the ERAS program impacts specific complications over time at our institution. In our study the ERAS society’s guidelines for liver surgery have shown a significant reduction in general postoperative complications, although the impact on surgery-related complications has not been as pronounced. Infectious related complications have been reduced, while classic complications like anastomotic leakage did not change significantly. However, a modest decline was observed in anastomotic leakage. Furthermore, a substantial decrease in ascites was observed within the ERAS group. This finding suggests the potential impact of the ERAS program on postoperative complications. The study also found that the type of surgery appears to be well balanced. The impact of these interventions on the outcomes is a plausible consideration. However, it is important to note that patient characteristics or the underlying diagnosis (malignant or benign) may introduce potential biases. However, further analysis is necessary to make a conclusive assessment. Specifically, the analysis should include an examination of the operating techniques and the extent of surgery.

There have been no significant changes in intraoperative techniques over the course of the observed period. As evidenced by our cohort data, the distribution of open, laparoscopic, and robotic surgery did not differ significantly between the pre-ERAS and ERAS groups. Consequently the surgical techniques did not change over time.

Previous studies reported on surgical and non-surgical complications with heterogeneous data^13–16^. Clark et al. performed a cohort study of 126 patients who underwent liver resection (Non-ERAS *n* = 73; ERAS *n* = 53)^14^. However, they did not find significant differences between individual complications. For example SSI 1, ileus, bile leak, renal failure, thrombosis or pulmonary embolism, septic shock, pneumonia were equally distributed between both groups. Note, this study was done in a relatively small cohort study and they did not follow the ERAS guidelines^14^. Another cohort study of Van Dam et al. reported on 161 patients who underwent liver resection (Non-ERAS *n* = 100; ERAS *n* = 61), while they found significantly more wound infections in the ERAS group^15^. For all other complications, like bile leakage, liver failure, abscess or non-surgical complications including pneumonia, delayed gastric function no significant effect was shown^15^. Note, they used a modified ERAS protocol from colorectal surgery for liver surgery. No difference in single complications was found by two other cohort studies which might be due to low sample size^13,16^. Strikingly, we could show a significant decrease in non-surgical complications which was mainly driven through a significant reduction in infectious complications (Non-ERAS 27.6% vs. ERAS 16.3%, *p* = 0.033), specifically a reduction in both wound infections (Non-ERAS 10.3% vs. ERAS 4.7%, p = < 0.055) and urinary tract infections (Non-ERAS 5,7% vs. ERAS 0%, p = < 0.001). From our point of view, this makes perfect sense. Improved wound healing can certainly be explained by early oral food and energy intake. The reduction in urinary tract infections and also urinary retention (Non-ERAS 2.4% vs. ERAS 0%, *p* = 0.063) can be explained by the early removal of the indwelling catheter. For the appearance of deep venous thrombosis we could not show a significant decrease but a slight trend toward the ERAS group is present (Non-ERAS 2.3% vs. ERAS 0.8%, *p* = 0.265). This might be due to the strict adherence to pre and postoperative prophylaxis even though this was also our policy before the ERAS implementation. An individual analysis of which individual components, e.g. pain management or mobilization, exactly led to a reduction was not possible within this study. We were surprised by the increased number of pulmonary complications in the ERAS group, even though these were not significant. This was mainly due to pleural effusions, although strictly following a restrictive fluid management intra- and postoperatively at our institution as it is required by the ERAS guidelines. Pneumonia was not more present in the ERAS group, which is consistent with previous studies. However, we would have expected a reduction in the ERAS group due to the early mobilization there. Even though pulmonary complications occurred more often in the ERAS group still the total amount of pulmonary complications were less than a recent study on pulmonary complications in hepatectomy, where pulmonary complications reached up to 13%^17^.

The CUSUM analysis (Fig. 1a-c) gives a good overview of the development of complications over time and shows changes in the process of ERAS implementation. To the best of our knowledge, no such analysis of complications in the ERAS program has been conducted to date. A notable decline in the incidence of non-surgical complications and the total amount of complications was observed in the ERAS cohort. For surgical complications, no clear trend was observed between non-ERAS and ERAS patients. Overall, the results of the CUSUM analysis also underline the observations of the statistical analysis. As with the development of compliance and length of stay, a decrease in complications can be observed over the period of the analysis.

Our findings indicate that adherence to the program experiences a consecutive increase from 62.5 to 72.5% over 4 years (Suppl. 2), while statistical significance has not been demonstrated. Also a decrease in length of hospital stay was observed (Suppl. 2). Initially, consistent introduction and implementation of individual components of the ERAS program are necessary, which may take some time. Once the program becomes firmly established in practice, adherence increases logically^18^.

The limitations of this study are primarily rooted in its observational nature with no randomization. To mitigate these constraints, PSM was employed. However, the considerable disparity in the size between the Non-ERAS and ERAS groups presents inherent challenges for direct comparisons. Although PSM was utilized to adjust for differences between the groups and to improve comparability, it is important to acknowledge that PSM, while effective in reducing bias, cannot substitute for randomization nor guarantee perfectly balanced groups. The study aimed to investigate specific complications, but the analysis was hampered by the relatively small size of the Non-ERAS cohort, making it difficult to apply the analysis with full effectiveness.

## Conclusion

The ERAS guidelines for liver surgery have shown a significant reduction in general postoperative complications, while the effects on surgical complications could not be demonstrated. Specifically, infection-related complications decreased, while classic complications such as anastomotic leakage did not change. Interestingly, the effect of the ERAS program improved continuously over time, which is why continuous work on the patient over years is required. Although our results provide valuable insights, randomized trials are needed to improve the evidence base and confirm the present results.

## Electronic supplementary material

Below is the link to the electronic supplementary material.

Supplementary Material 1

Supplementary Material 2

## Data Availability

The datasets used and/or analyzed during the current study are available from the corresponding author upon reasonable request.
