# Supplementary figures and images for: Enhanced recovery after surgery society’s recommendations for liver surgery reduces non surgical complications
Source: Sci Rep. 2025 Jan 29;15:3693. doi: 10.1038/s41598-025-86808-z (PMC11779921; doi:10.1038/s41598-025-86808-z)

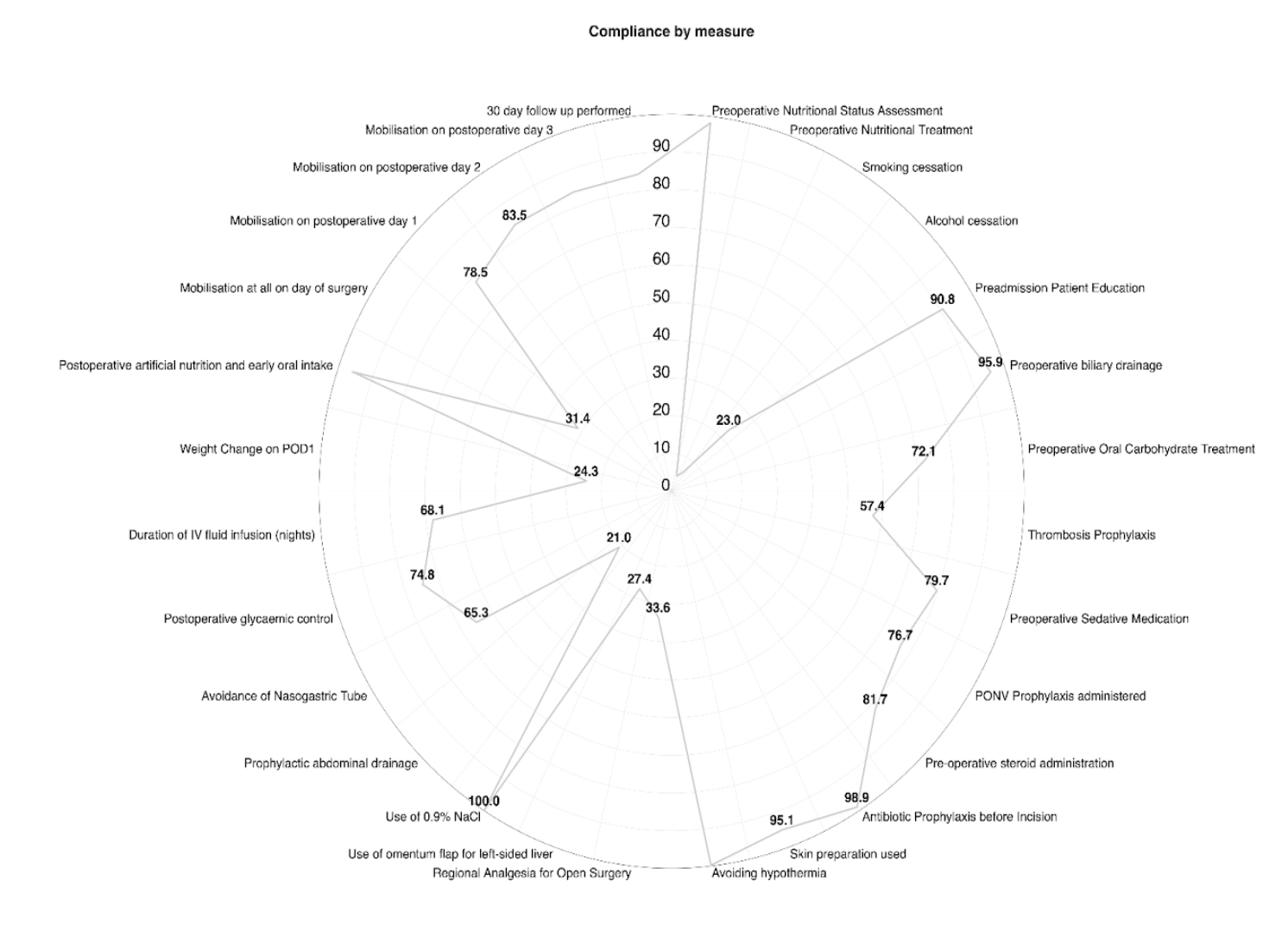

Supplement: Supplementary file 1 — Supplementary Material 1 [file 41598_2025_86808_MOESM1_ESM.jpg]

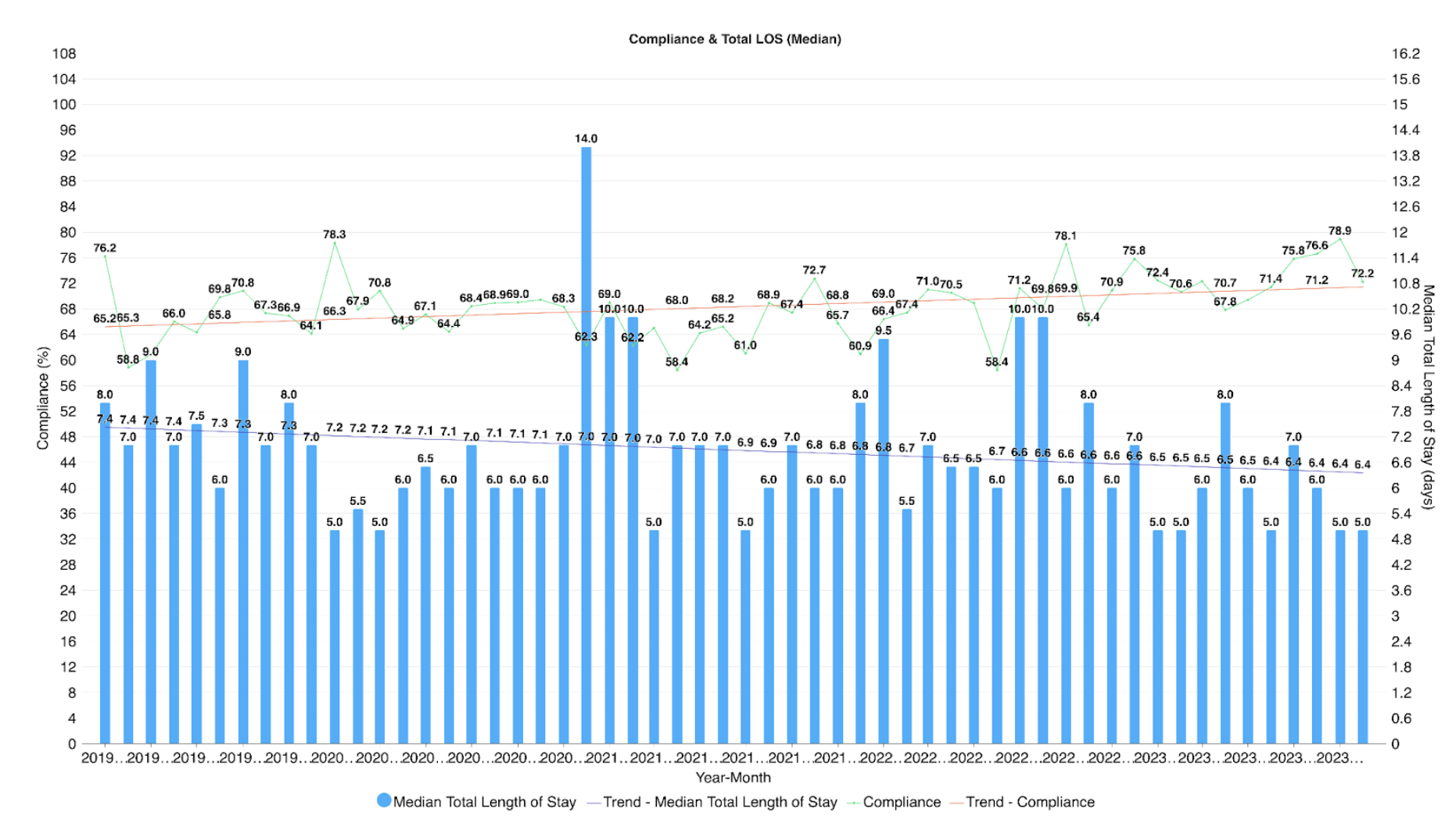

Supplement: Supplementary file 2 — Supplementary Material 2 [file 41598_2025_86808_MOESM2_ESM.jpg]
